# Supplementary material for: Single virus fingerprinting by widefield interferometric defocus-enhanced mid-infrared photothermal microscopy
Source: Nat Commun. 2023 Oct 20;14:6655. doi: 10.1038/s41467-023-42439-4 (PMC10589364; doi:10.1038/s41467-023-42439-4)
Supplement: Supplementary file 1 — Supplementary Information [file 41467_2023_42439_MOESM1_ESM.pdf]

SUPPLEMENTARY INFORMATION

Single virus fingerprinting by widefield interferometric defocus-enhanced mid-infrared photothermal microscopy

Qing Xia[1], Zhongyue Guo[2], Haonan Zong[1], Scott Seitz[3], Celalettin Yurdakul[1], M. Selim Ünlü[1], Le Wang[1], John H. Connor[3,]* and Ji-Xin Cheng[1,2,4,]*

[1] Department of Electrical and Computer Engineering, Boston University, Boston, Massachusetts 02215, United States;
[2] Department of Biomedical Engineering, Boston University, Boston, Massachusetts 02215, United States;
[3] Department of Microbiology and National Infectious Diseases Laboratories, Boston University School of Medicine, Boston, Massachusetts 02118, United States;
[4] Photonics Center, Boston University, Boston, Massachusetts 02215, United States.
* Correspondence: jxcheng@bu.edu (J.X.C.), jhconnor@bu.edu (J.H.C)

Summary

Number of Pages: 16

Pages 2-4: Supplementary Notes 1-8
Page 5: Supplementary Table 1
Pages 6-15: Supplementary Figs. 1-10
Page 16: Supplementary References

Supplementary Note 1. Simulation of the temperature rise.

To better understand the photothermal process, a theoretical model was built to solve the temperature difference between hot and cold states. This model was developed using COMSOL based on our previous work[1]. The time-dependent thermal diffusion process can be simulated via the heat-transfer-in-solids module in COMSOL Multiphysics[2]. To calculate the heat dissipation, a heat source term $\boldsymbol{Q(t)}$ is defined as below:

$$C_p\rho\frac{\partial T}{\partial t} + \nabla\cdot(-k\nabla T) = Q(t) \qquad (1)$$

where T is the temperature, t is the time, C_p is the heat capacity, ρ is the density, and k is the thermal conductivity of the material in the system.

In this simulation, a 200 nm PMMA bead was sitting on the silicon substrate in air. The IR heating beam size is 24.6 µm by 30 µm and the power is 40 mW measured from the experiments. Both the initial temperature and the simulation boundary were assumed to be 298 K. The heat source was defined as the domain of the PMMA bead. Heat convection was not considered in this simulation. By solving the equation (1), the temperature rise distribution of the bead under single IR pulse heating was simulated via COMSOL 6.0. Supplementary Fig. 2a shows the temperature profile of the system. The calculated ΔT on the single bead is ~80 K, integrated from the pulse width of single probe pulse, which is ~129 ns (Supplementary Fig. 2b).

Supplementary Note 2. Interferometric defocus-enhanced MIP signal for PMMA beads with different sizes.

For PMMA beads with different sizes, the defocus curves of interferometric and MIP contrasts have different shapes. The resulting ΔZ between the maximum interferometric and MIP contrasts varies with the particle size. Similar mechanism of interferometric defocus-enhanced MIP signal was also validated for D = 100 nm and D = 150 nm PMMA (Supplementary Fig. 3, a and b), where the simulated focal plane difference ΔZ is found to be 200 nm and 300 nm, respectively. While for big nanoparticles with diameter of 350 nm, 400 nm and 500 nm, the increased particle size results in a noticeable change of the defocus curve in interferometric imaging[3] (Supplementary Fig. 3, c to e), and the resulted ΔZ was estimated to be < 100 nm. The simulated interferometric and MIP images for D = 500 nm PMMA show similar maximum at Z = 400 and 500 nm, and decreased contrasts at Z =0 nm (Supplementary Fig. 3f). The experimental defocus curve and images for D = 500 nm PMMA also match the simulation results very well (Supplementary Fig. 3, g to h). It indicates that defocusing does not help MIP enhancement for big particles with diameter larger than 300 nm. Since most viruses vary in diameter from 20 nm to 300 nm, defocusing especially works well for small nanoparticles with similar size of single virus, which provides WIDE-MIP as a promising tool for single virus analysis.

Supplementary Note 3. Detection limit of WIDE-MIP imaging.

To demonstrate high-speed widefield photothermal detection of small nanoparticles, we performed WIDE-MIP imaging of both D = 200 nm and D = 100 nm PMMA beads. The defocused interferometric images of beads in air are shown in Supplementary Fig. 4a (D = 200 nm PMMA) and Supplementary Fig. 4b (D = 100 nm PMMA). WIDE-MIP images of both types of PMMA beads showed high contrast at 1728 cm^{-1}, indicating C=O vibration in PMMA (Supplementary Fig. 4, c and d), while no contrasts were observed at the off-resonance 1800 cm^{-1} (Supplementary Fig. 4, e and f). The WIDE-MIP images were acquired with the signal averaged for 2.36 s, showing good SNR of ~ 87 for 200 nm PMMA and SNR of ~14 for 100 nm PMMA.

Supplementary Note 4. Spatial resolution of WIDE-MIP imaging.

To evaluate the spatial resolution of WIDE-MIP, we first measured the interferometric contrast profile across one single 200 nm PMMA bead at different focal planes. The Gaussian fitted full width at half maximum (FWHM) along the horizontal axes is 278 nm at the interferometric focus Z = 0.4 µm (Supplementary Fig. 5a), which is consistent with the theoretical resolution of the interferometric imaging system. In WIDE-MIP, the interferometric image is defocused at Z = 0 µm, the FWHM along the particles is 404 nm, which is a little larger than the resolution of the interferometric system (Supplementary Fig. 5b). Thus, the spatial resolution is 417 nm, measured as FWHM of particles in the WIDE-MIP image captured at the defocus plane of Z = 0 µm (Supplementary Fig. 5c). In addition, WIDE-MIP system allows depth resolved measurement, the depth of focus for the imaging system is 503

nm, measured as FWHM of the MIP contrast profile in WIDE-MIP image from Z = -1 μm to Z = 1 μm (Supplementary Fig. 5d).

Supplementary Note 5. Atomic force microscope analysis of single viruses.

Size and morphology characterizations of single vaccinia viruses (VACV) were performed on an atomic force microscope (AFM) (NanoWizard 4 XP, Bruker Nano) in tapping mode (Supplementary Fig. 6a). AFM analysis further confirmed the single VACV particle 1 on the silicon substrate with a brick-shaped size of 280 × 350 nm (Supplementary Fig. 6b) that is consistent with the reported viral shape and dimensions [4, 5] VACV particle 2 looks like a denatured rather than intact virion (Supplementary Fig. 6c). It is highly likely happened during the fixation process.

Supplementary Note 6. Peak assignments of viral WIDE-MIP spectra.

The assignments of the chemical components were validated by the pure protein, DNA and RNA film samples (Supplementary Fig. 8). The dominant two peaks in the spectrum of pure BSA are contributed by the amide I band at 1650 cm^{-1} and the amide II band at 1550 cm^{-1}, indicating the vibrations in the protein[6]. For the assignments of base residues in the nucleic acids, according to the literature[7], the peaks at 1656 cm^{-1} and 1604 cm^{-1} are assigned to the NH_2 bending and C=N streching vibrations in adenine (A) residue. The peaks at 1680 cm^{-1} and 1640 cm^{-1} are assigned to the NH in-plane deformation vibration and C=O and C=C stretching vibrations in uracil (U) residue, respectively. The peaks at 1656 cm^{-1} and 1580 cm^{-1} are assigned to the C_4=O stretching vibration and ring stretching vibration in thymine (T) residue, respectively. The peak at 1604 cm^{-1} is assigned to the In-plane ring vibrations in cytosine (C) residue. The peak at 1692 cm^{-1} is assigned to the C=O stretching vibration and NH_2 scissoring vibration in guanine (G) residue. Although the peak assignments of base residues in literature show ~ 10 cm^{-1} higher than our results, we attribute this small difference to the different sample state, testing environment and instruments. The peak at ~ 1725 cm^{-1} is assigned to the C=O stretching vibration in viral lipids[8].

To further demonstrate the accuracy of MIP spectra for virus fingerprinting, we provided the biochemical components and the FTIR spectrum of pure VZV powder for comparison (Fig. 5l). For a VZV, it has a lipid-rich envelope derived from cellular membranes, within which viral glycoproteins are inserted[9]. Within the VZV, there are ~ 125-kb linear double-stranded DNA genome and ~ 3000 proteins[10]. Notably, three envelope proteins, namely glycoprotein B, glycoprotein H, and glycoprotein L, have been identified as essential VZV proteins forming the core fusion complex[9]. These proteins have known 3D structure, and a significant proportion of their secondary structures consist of β-sheets, with turn structures also present[11]. The results in Fig. 5 showed that WIDE-MIP can accurately identify VZV viruses and reveal T residue vibrations in viral DNA, lipids and enriched β sheet components in VZV viral proteins, which are consistent with the biochemical components in VZV and FTIR spectrum of VZV powder.

Supplementary Note 7. Comparison between fluorescence-detected mid-infrared photothermal microscopy (F-MIP) and WIDE-MIP.

While F-MIP imaging offers a larger modulation depth[12, 13], WIDE-MIP offers a distinct advantage in detecting bionanoparticles that have very low levels of expressed fluorescence tags.

One of the key requirements for F-MIP is a robust and high-quality fluorescence signal. In this regard, it is essential to highlight the difference in fluorescence probe labeling quantity between our previous study conducted by Yi Zhang et al.[12] and the current work. In Yi Zhang et al's work, high concentrations of commercial chemical dyes (10 µM Nile Red or Rhodamine) were used for labeling high-content biological components, such as proteins in cells. While in this work, both VACV and VSV viruses were expressed with an enhanced green fluorescent protein (eGFP) envelope. The expressed eGFP was fused to the VSV G protein, where each VSV contains ~ 1,200 molecules of the G protein on the viral surface[14]. With the formed G protein and G-eGFP fusion protein heterodimers, there are ~ 600 eGFP molecules on the surface of a single virus. Comparing the size of eGFP (2.4 × 4.2 nm) to that of the VSV virus (80 × 180 nm), we estimate that only 1.3% of a single VSV virus consists of eGFP. Thus, due to the lower content of total fluorescence probes in this study, the resulting fluorescence intensity is significantly weaker compared to Yi Zhang et al's work. Consequently, the reduced fluorescence

intensity poses a challenge when attempting to implement fluorescence-based MIP imaging of single viruses in this work.

To demonstrate it, we used the same camera (FLIR, Grasshopper3GS3-U3-51S5M) as in Yi Zhang et al's work to perform the fluorescence imaging of VACVs expressing eGFP, and tried the same paraments with a camera exposure time of 50 ms and a gain of 20 dB. However, these settings failed to capture the fluorescence of single viruses. We increased the exposure time to 500 ms for imaging aggregated viruses, while using the exposure time of ~ 5 s and maximum gain setting for single viruses (Supplementary Fig. 9a to d). Although the photobleaching of aggregated virus showed a similar level as Yi Zhang et al's work (< ~ 10%, Supplementary Fig. 9e), severe photobleaching was observed in single viruses (> ~ 95%, Supplementary Fig. 9f). This photobleaching of single viruses limits the detection of photothermal modulation and acquisition speed. Considering that the MIP signal relies on the difference in fluorescence intensity between the IR-on (hot) and IR-off (cold) states, this severe bleaching at the single-virus level further compromises the reliability of F-MIP.

On the other hand, scattering-based imaging offers certain advantages, particularly in terms of the photon budget. When compared to fluorescence imaging, scattering-based techniques allow for shorter camera exposure times, higher full well capacity, and reduced saturation issues. In Yi Zhang et al's work, a CMOS camera (FLIR, Grasshopper3GS3-U3-51S5M) with a full well depth of ~ 10,000 and a frame rate of 20 Hz was used for wide-field FMIP imaging. In this work, we employed a camera with a frame rate of 1270 Hz and a full well capacity of 2 million wells (Q-2HFW, Adimec). This choice ensured that sufficient probe photons were received at each pixel, enhancing the quality of the scattering signal. Additionally, the interferometric geometry further enhanced the weak scattering signal of single viruses.

Taking into account these limitations and technical considerations, F-MIP imaging is not suitable for the detection of single viruses. Instead, we focused on fluorescence-guided MIP analysis of single viruses. In this work, we first collected and analyzed the fingerprint spectra of eGFP-virus samples (VACV and VSV) and performed co-localization of fluorescence imaging and MIP imaging to demonstrate the accurate identification of viruses using the WIDE-MIP technique (Figure 4). However, for the analysis of actual virus samples, label-free methods may be more suitable for diagnostic purposes. Thus, we further performed MIP imaging and fingerprint spectra of unlabeled pure VZV viruses to achieve label-free detection of single viruses (Figure 5).

Supplementary Note 8. Potential application for rapid quality control of viral vectors.

We further demonstrated high-speed chemical imaging of single VACV by reducing the acquisition time to 0.32 s per image per wavenumber of single viruses and the SNR of one single VACV is ~ 4 within the field of view (FoV) of 24 by 24 μm (Supplementary Fig. 10). In comparison, the image acquisition time of one single virus is 46.4 s at the FoV of ~2.3 by 2.3 μm in previous work [15]. Together, WIDE-MIP microscopy provides ~1000-fold higher throughput, enabling fingerprinting of viral vectors for quality control.

To use this method, the viral vector products can be prepared on a silicon substrate following the sample preparation protocol and then imaged using WIDE-MIP. The substrate will then be taken for WIDE-MIP imaging and get the fingerprints of the particles from the products. For fingerprint region from 1500 cm^{-1} to 1750 cm^{-1}, fingerprinting at each FoV will take at least 8.32 s with the scanning step at 10 cm^{-1}. Quality control results can be obtained by comparing the fingerprints of the products with those from the standard viral vector, followed by spectral analysis of viral proteins and viral nuclei acids to determine stability, purity, and integrity. Additionally, WIDE-MIP can advance the development of new viral vectors by facilitating their characterization and optimization.

Supplementary Table 1. Comparison of detection limit of MIP imaging.

	Substrate	Sample	SNR	Throughput (beads/min)
**A** [2]	Glass	100 nm PS	~ 70	1
**B** [16]	CaF_2	100 nm PS	~ 10-50	1.5
**C** [15]	CaF_2	100 nm PMMA	~ 13	3
**This work**	Silicon	100 nm PMMA	~ 21	> 3000

PS: polystyrene. PMMA: polymethyl methacrylate. SNR: signal-to-noise ratio.

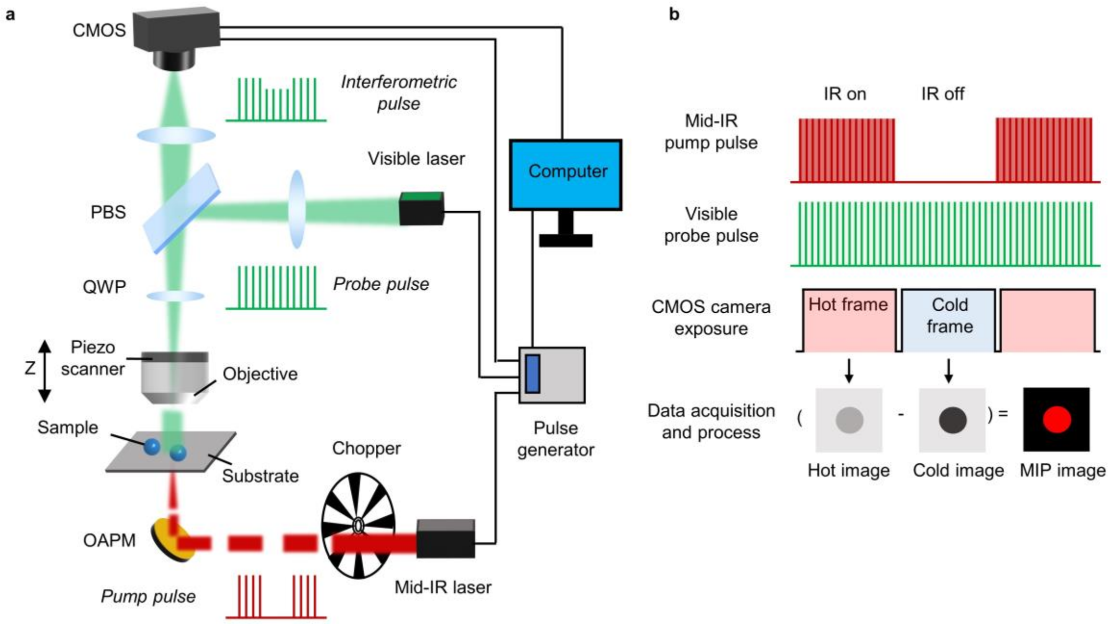

Supplementary Fig. 1 WIDE-MIP microscope setup and signal synchronization. (a) Schematic of WIDE-MIP microscope. The IR pump beam was generated by a tunable (from 1400 to 1800 cm^{-1}) mid-IR laser operating at 20 kHz repetition rate with a ~20 ns pulse duration, which was further modulated by an optical chopper. The visible probe was provided with a pulsed 520 nm nanosecond laser with a pulse duration of 129 ns. The interferometric scattering was recorded by a 2 million well-depth camera. A delay pulse generator is used to synchronize the pump pulse, probe pulse and camera. PBS: Polarizing beam splitter, OAPM: Off-axis parabolic mirror, QWP: quarter-wave plate, CMOS: complementary metal-oxide semiconductor. (b) Illustration for the synchronization and data acquisition of WIDE-MIP microscopy. To synchronize and acquire data for WIDE-MIP microscopy, the delay pulse generator was triggered by the output signal from the nanosecond IR laser. The oscilloscope was used to monitor the IR and visible pulses through a Mercury-Cadmium-Telluride detector and a photodiode, respectively. The IR pulses were modulated to a 50% duty cycle by the optical chopper. The camera trigger signal delay was adjusted to capture both IR on (hot) and IR off (cold) frames. The MIP contrast was generated by the subtraction of hot and cold frames.

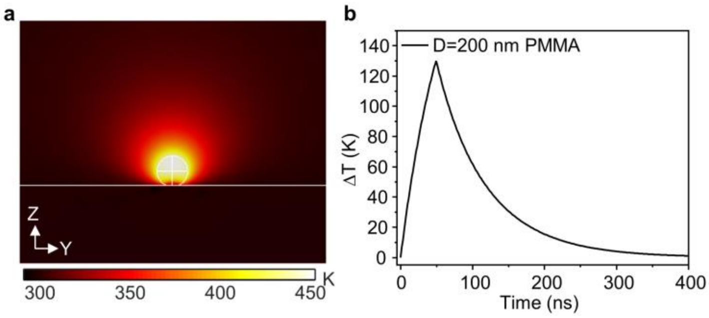

Supplementary Fig. 2 Simulated temperature rise of a 200 nm PMMA bead under single IR pulse heating. (a) Temperature distribution of a 200 nm PMMA bead on the silicon substrate heated by a single IR pulse. Time is at 400 ns after the rising edge of the IR pulse. (b) Simulation results of thermodynamic properties of the heated PMMA bead.

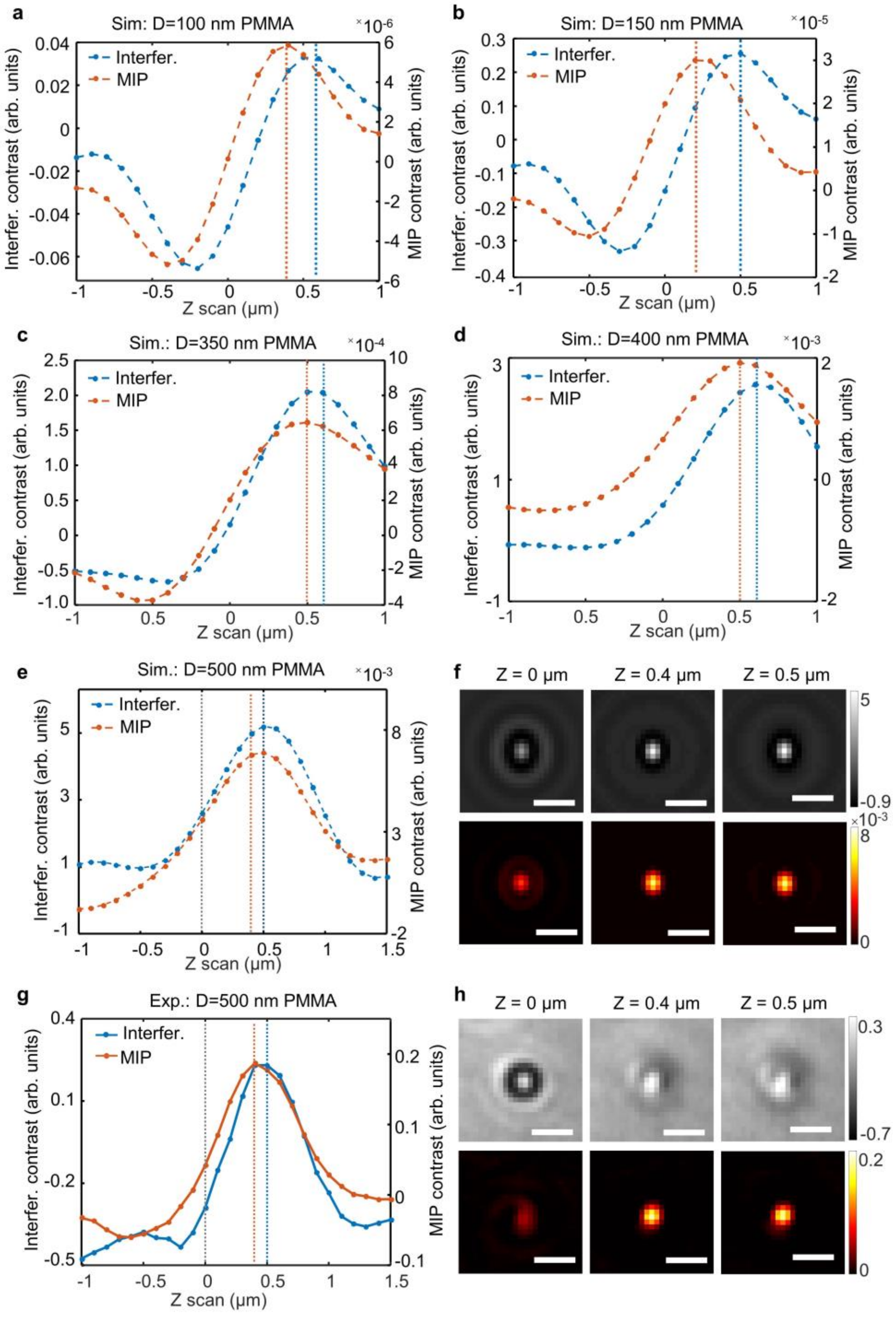

Supplementary Fig. 3 Interferometric defocus enhancement of MIP contrasts of PMMA beads with different sizes. Simulated defocus curves of interferometric and MIP contrasts of single (a) D = 100 nm, (b) D = 150 nm, (c) D = 350 nm, (d) D = 400 nm, (e) D = 500 nm PMMA bead. (f) Simulated interferometric and MIP images at Z = 0 μm, Z = 0.4 μm, and Z = 0.5 μm. FoV: 3 μm by 3 μm. Scale bars: 1 μm. (g) Experimental defocus curves of interferometric and MIP contrasts for D = 500 nm PMMA beads. (h) Experimental interferometric and MIP images at Z = 0 μm, Z = 0.4 μm, and Z = 0.5 μm. FoV: 3 μm by 3 μm. Red dot lines indicate the maximum MIP contrasts. Blue dot lines indicate the maximum interferometric contrasts. Grey dot lines indicate Z = 0 μm. Power before the objective: pump: 48 mW at 1728 cm^{-1}, probe: ~1 mW. Acquisition time: 2.36 s per image. Objective piezo scanner, Piezosystemjena, MIPOS 100, Z-axis scanning step: 100 nm. FoV: field of view. D: Diameter. Interfer.: Interferometric. ΔT was set to 1 K for a simplified simulation.

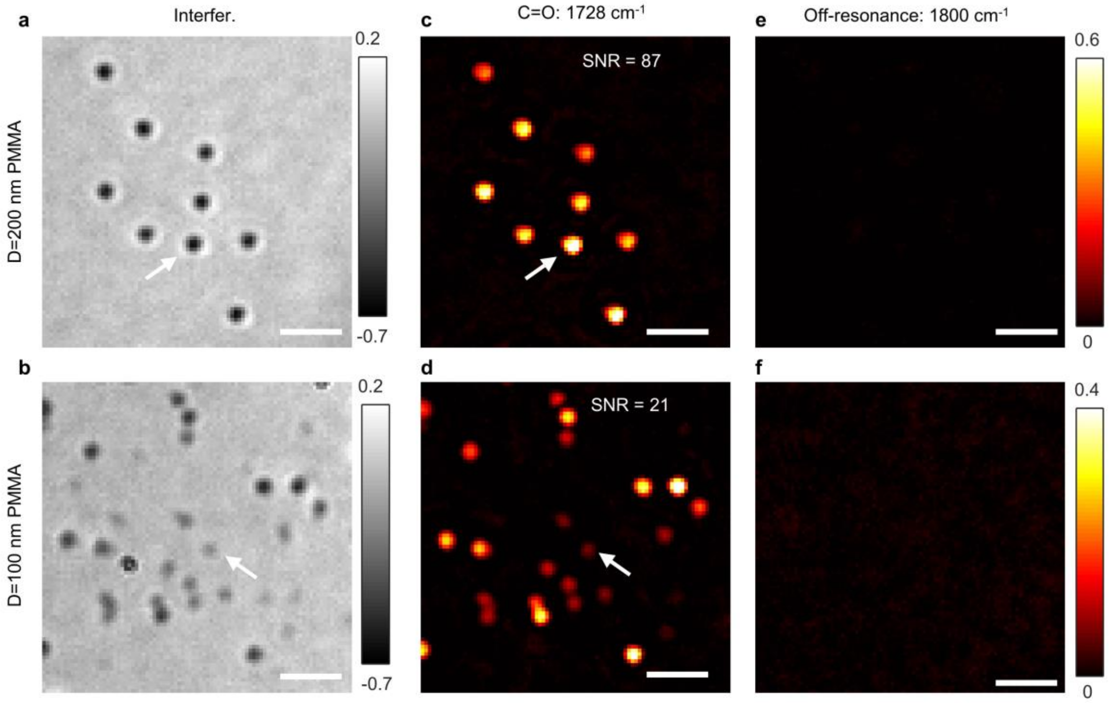

Supplementary Fig. 4 Detection limit of WIDE-MIP imaging. Defocused interferometric scattering image of (a) D = 200 nm and (b) D = 100 nm PMMA beads. (c, d) MIP image of the same area with the pump at 1728 cm^{-1}. (e, f) Off-resonance image showed no contrast. Scale bars: 2 μm. Power before the objective: pump: 31.4 mW at 1728 cm^{-1}, 32.2 mW at 1800 cm^{-1}, probe: ~1 mW. Image acquisition time: 2.36 s per image. The MIP intensities are normalized by the IR power. The larger signals in panel (d) are from particle aggregates.

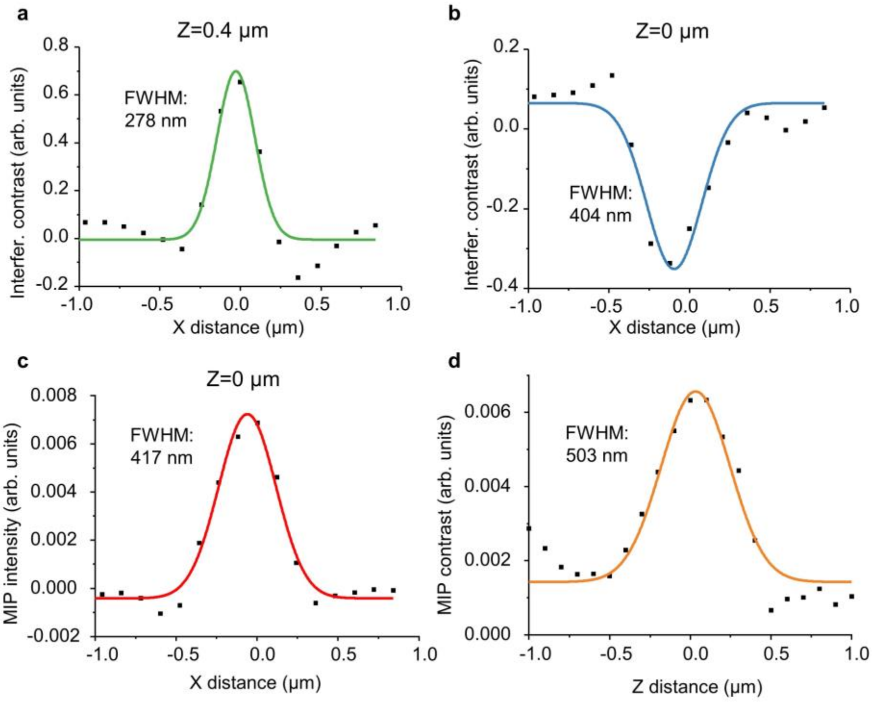

Supplementary Fig. 5 Spatial resolution of WIDE-MIP imaging. (a, b) Horizontal cross-sectional profiles of interferometric scattering image across one single bead (shown in Fig. 2f) at (a) Z = 0.4 µm and (b) Z = 0 µm. The Gaussian fitted FWHMs are 278 and 404 nm. (c) Horizontal cross-sectional profiles of MIP image across the same bead (shown in Fig. 2g) at Z = 0 µm. The Gaussian fitted FWHM is 417 nm. (d) Axial cross-sectional profiles of MIP image across the same bead (shown in Fig. 2g) at Z = 0 µm. The depth of focus for MIP imaging is 503 nm, calculated from the Gaussian fitted FWHM.

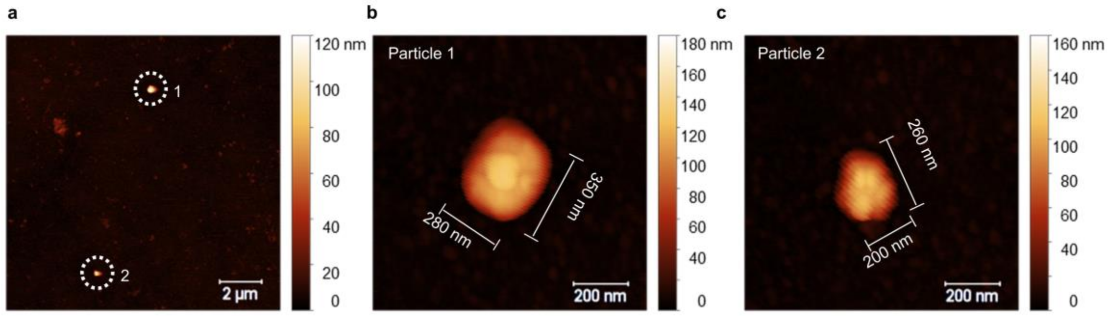

Supplementary Fig. 6 AFM analysis of size and shape of a single VACV. (a) AFM characterization of a single VACV virus in air. (b) and (c) are zoom-in AFM images of single viruses labeled with dotted circles in (a).

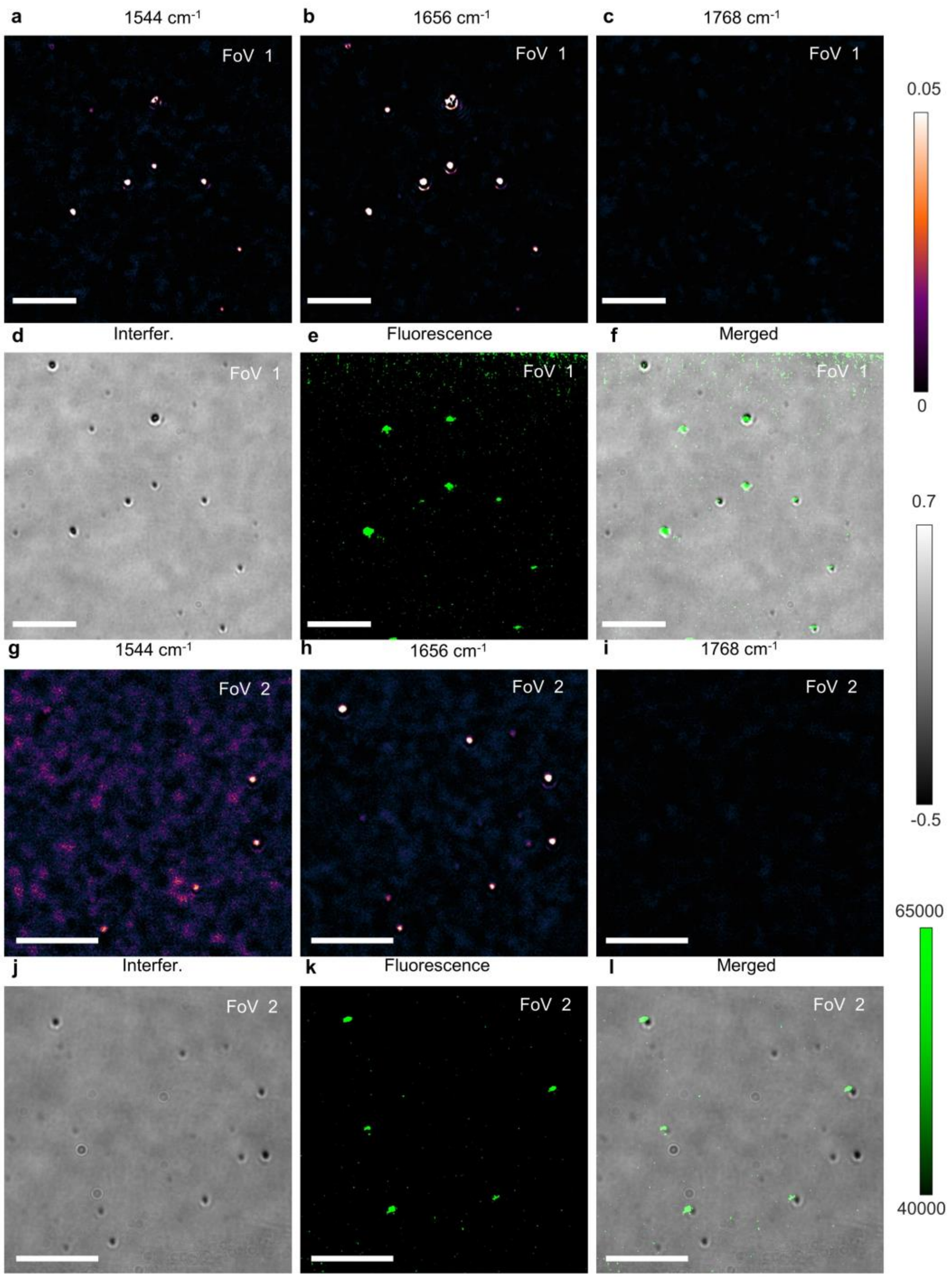

Supplementary Fig. 7 Fingerprinting detection of single VSVs. (a) Amide II bond-selective image of single VSV viruses with the pump at 1552 cm^{-1} of FoV 1. (b) Amide I bond-selective image of the same with the pump at 1656 cm^{-1}. (c) Off-resonance image showed no contrast. (d) Defocused interferometric scattering, (e) fluorescence and (f) merged images of the same area. (g) Amide II bond-selective image of single VSV viruses with the pump at 1552 cm^{-1} of FoV 2. (h) Amide I bond-selective image of the same with the pump at 1656 cm^{-1}. (i) Off-resonance image showed no contrast. (j) Defocused interferometric scattering, (k) fluorescence and (l) merged images of the same area. Scale bars: 10 μm. Power before the objective: pump: 29.1 mW at 1552 cm^{-1}, 34.5 mW at 1656 cm^{-1}, 35.8 mW at 1768 cm^{-1}, probe: ~1 mW. Image acquisition time: 2.36 s per wavenumber. The MIP spectrum is normalized by the IR power. FoV: field of view.

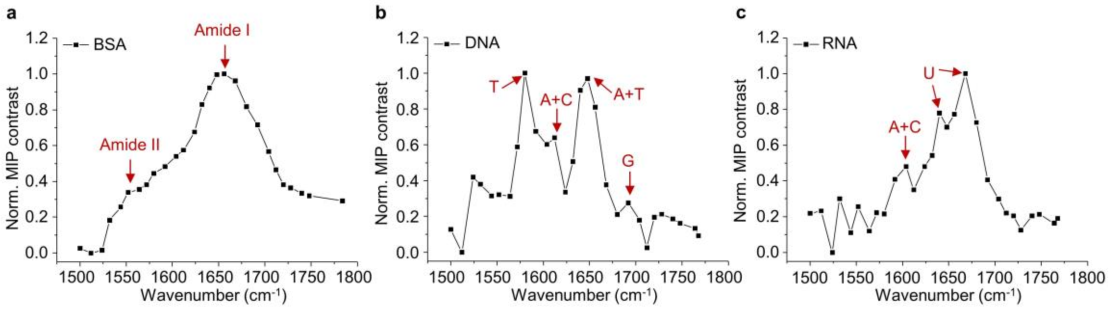

Supplementary Fig. 8 WIDE-MIP spectra of pure chemicals. WIDE-MIP spectra of (a) dried pure protein (BSA) film, (b) dried pure DNA film (cDNA of melanoma cell), and (c) dried pure RNA film (ssRNA of T24 cell). The MIP spectra are normalized by the IR power under each wavenumber.

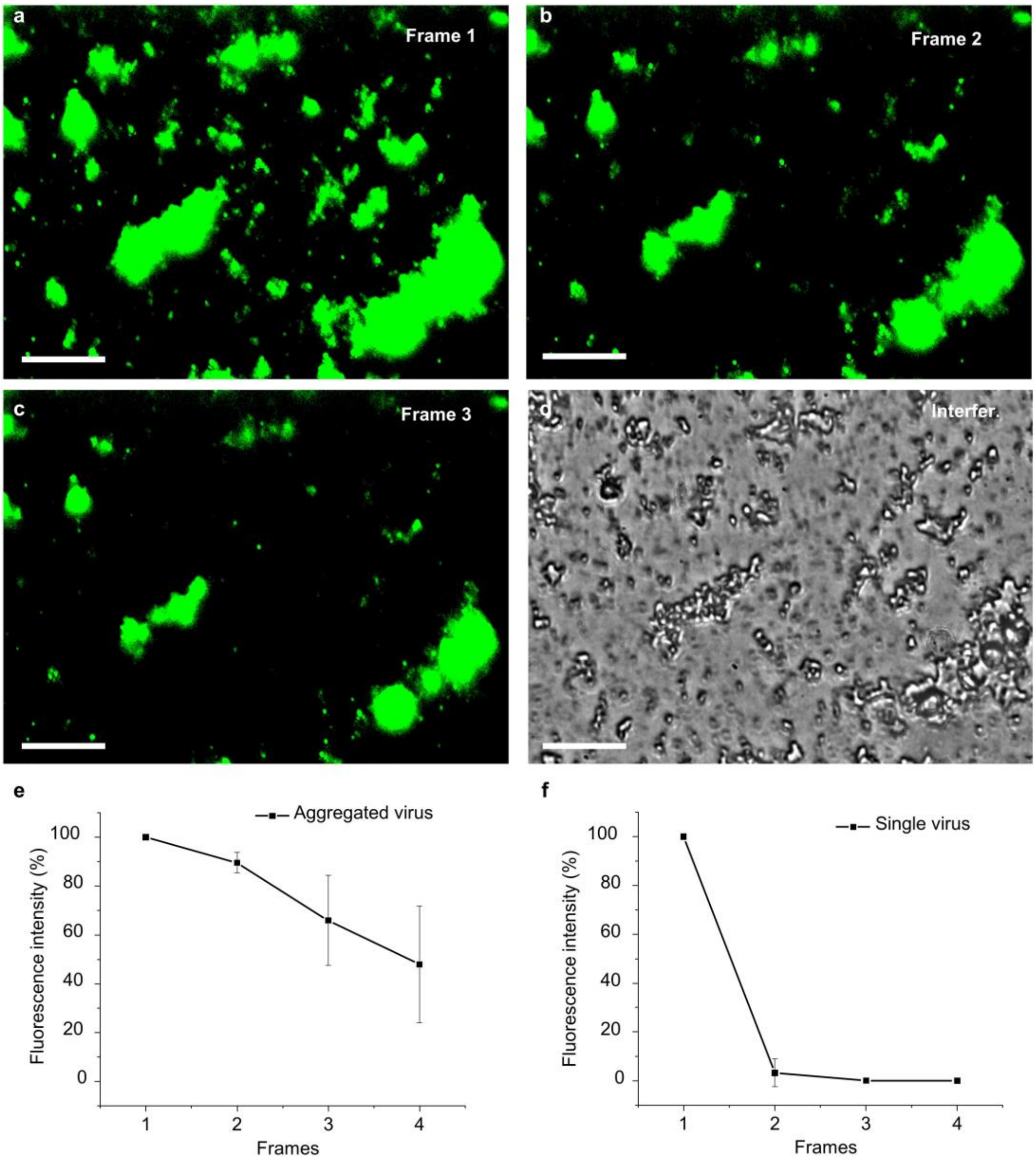

Supplementary Fig. 9 Photobleaching analysis of single VACVs. (a-c) Continuous fluorescence imaging of VACVs from frame 1 to frame 3. (d) Defocused interferometric scattering image of the same area in (a-c). Scale bars: 10 μm. Image acquisition time: 5 s/image. Fluorescence intensity of (e) aggregated viruses (n =15) and (f) single viruses (n = 20) in the field of view. Error bars stand for the standard deviations of mean fluorescence intensity.

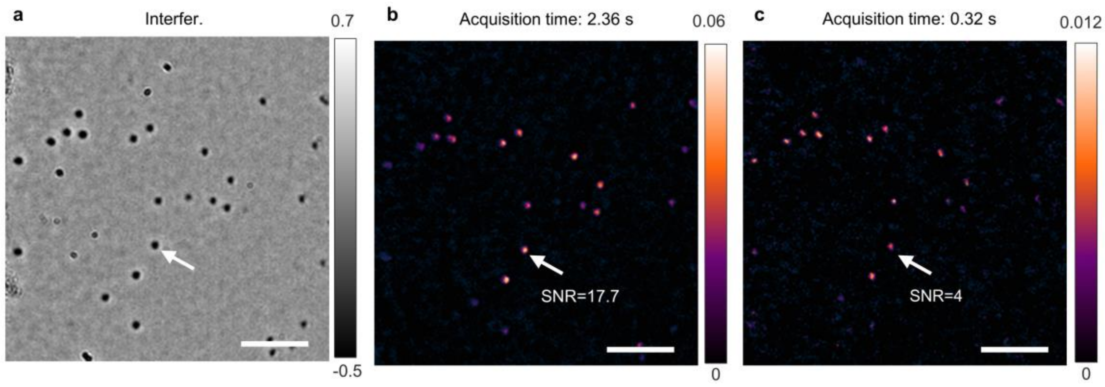

Supplementary Fig. 10 High speed chemical imaging of single VACVs. (a) Defocused interferometric scattering image of single VACV viruses. Amide I bond-selective image of the same area with the image acquisition times of (b) 2.36 s and (c) 0.32 s. The pump beam wavenumber is set to 1656 cm^{-1}. Power before the objective: 34.5 mW at 1656 cm^{-1}, probe: ~1 mW. Scale bars: 5 μm.

Supplementary References

1. Zong H, Yurdakul C, Bai Y, Zhang M, Unlu MS, Cheng JX. Background-suppressed high-throughput mid-infrared photothermal microscopy via pupil engineering. *ACS Photonics* **8**, 3323-3336 (2021).
2. Li Z, Aleshire K, Kuno M, Hartland GV. Super-resolution far-field infrared imaging by photothermal heterodyne imaging. *J. Phys. Chem. B* **121**, 8838-8846 (2017).
3. Trueb J, Avci O, Sevenler D, Connor JH, Unlu MS. Robust visualization and discrimination of nanoparticles by interferometric imaging. *IEEE J. Sel. Top. Quantum Electron.* **23**, 394-403 (2017).
4. Cyrklaff M, Risco C, Fernandez JJ, Jimenez MV, Esteban M, Baumeister W, *et al.* Cryo-electron tomography of vaccinia virus. *Proc. Natl. Acad. Sci. U.S.A.* **102**, 2772-2777 (2005).
5. Malkin AJ, McPherson A, Gershon PD. Structure of intracellular mature vaccinia virus visualized by in situ atomic force microscopy. *J. Virol.* **77**, 6332-6340 (2003).
6. Barth A. Infrared spectroscopy of proteins. *Biochim. Biophys. Acta.* **1767**, 1073-1101 (2007).
7. Tsuboi M. Application of infrared spectroscopy to structure studies of nucleic acids. *Appl. Spectrosc. Rev.* **3**, 45-90 (1970).
8. Movasaghi Z, Rehman S, ur Rehman DI. Fourier transform infrared (FTIR) spectroscopy of biological tissues. *Appl. Spectrosc. Rev.* **43**, 134-179 (2008).
9. Zerboni L, Sen N, Oliver SL, Arvin AM. Molecular mechanisms of varicella zoster virus pathogenesis. *Nat. Rev. Microbiol* **12**, 197-210 (2014).
10. Wang W, Zheng Q, Pan D, Yu H, Fu W, Liu J, *et al.* Near-atomic cryo-electron microscopy structures of varicella-zoster virus capsids. *Nat. Microbiol.* **5**, 1542-1552 (2020).
11. Berman H, Henrick K, Nakamura H. Announcing the worldwide Protein Data Bank. *Nat. Struct. Biol.* **10**, 980 (2003).
12. Zhang Y, Zong H, Zong C, Tan Y, Zhang M, Zhan Y, *et al.* Fluorescence-detected mid-infrared photothermal microscopy. *J. Am. Chem. Soc.* **143**, 11490-11499 (2021).
13. Li M, Razumtcev A, Yang R, Liu Y, Rong J, Geiger AC, *et al.* Fluorescence-Detected Mid-Infrared Photothermal Microscopy. *J. Am. Chem. Soc.* **143**, 10809-10815 (2021).
14. Pol ANvd, Dalton KP, Rose JK. Relative neurotropism of a recombinant rhabdovirus expressing a green fluorescent envelope glycoprotein. *J. Virol.* **76**, 1309-1327 (2002).
15. Zhang Y, Yurdakul C, Devaux AJ, Wang L, Xu XG, Connor JH, *et al.* Vibrational spectroscopic detection of a sngle virus by mid-infrared photothermal microscopy. *Anal. Chem.* **93**, 4100-4107 (2021).
16. Pavlovetc IM, Podshivaylov EA, Chatterjee R, Hartland GV, Frantsuzov PA, Kuno M. Infrared photothermal heterodyne imaging: Contrast mechanism and detection limits. *J. Appl. Phys.* **127**, 165101 (2020).
